# Computed Tomography-Based Coronary Artery Calcium Score Calculation at a Reduced Tube Voltage Utilizing Iterative Reconstruction and Threshold Modification Techniques: A Feasibility Study

**DOI:** 10.3390/diagnostics13213315

**Published:** 2023-10-26

**Authors:** Shirin Habibi, Mohammad Akbarnejad, Nahid Rezaeian, Alireza Salmanipour, Ali Mohammadzadeh, Kiara Rezaei-Kalantari, Hamid Chalian, Sanaz Asadian

**Affiliations:** 1 Department of Radiology, School of Medicine, Iran University of Medical Sciences, Tehran 1449614535, Iran; habibi.shirin97@gmail.com; 2 Department of Radiology, Rajaie Cardiovascular Medical and Research Center, Iran University of Medical Sciences, Tehran 1449614535, Iran; mohamad.akn8@gmail.com (M.A.); nahid6069@yahoo.com (N.R.); asalmanipour@gmail.com (A.S.); a.mohammadzadeh@rhc.ac.ir (A.M.); rkkiara@gmail.com (K.R.-K.); 3 Department of Radiology, Cardiothoracic Imaging, University of Washington, Seattle, WA 98105, USA; hchalian@uw.edu

**Keywords:** coronary artery calcium score (CACS), effective dose (ED), iterative reconstruction (IR), Hounsfield unit (HU) threshold modification

## Abstract

Background: The coronary artery calcium score (CACS) indicates cardiovascular health. A concern in this regard is the ionizing radiation from computed tomography (CT). Recent studies have tried to introduce low-dose CT techniques to assess CACS. We aimed to investigate the accuracy of iterative reconstruction (IR) and threshold modification while applying low tube voltage in coronary artery calcium imaging. Methods: The study population consisted of 107 patients. Each subject underwent an electrocardiogram-gated CT twice, once with a standard voltage of 120 kVp and then a reduced voltage of 80 kVp. The standard filtered back projection (FBP) reconstruction was applied in both voltages. Considering Hounsfield unit (HU) thresholds other than 130 (150, 170, and 190), CACS was calculated using the FBP-reconstructed 80 kVp images. Moreover, the 80 kVp images were reconstructed utilizing IR at different strength levels. CACS was measured in each set of images. The intraclass correlation coefficient (ICC) was used to compare the CACSs. Results: A 64% reduction in the effective dose was observed in the 80 kVp protocol compared to the 120 kVp protocol. Excellent agreement existed between CACS at high-level (strength level = 5) IR in low-kVp images and the standard CACS protocol in scores ≥ 11 (ICC > 0.9 and *p* < 0.05). Increasing the threshold density to 190 HU in FBP-reconstructed low-kVp images yielded excellent agreement with the standard protocol in scores ≥ 11 (ICC > 0.9 and *p* < 0.05) and good agreement in score zero (ICC = 0.84 and *p* = 0.02). Conclusions: The modification of the density threshold and IR provides an accurate calculation of CACS in low-voltage CT with the potential to decrease patient radiation exposure.

## 1. Introduction

Coronary artery disease (CAD), followed by stroke and lower respiratory diseases, is the leading cause of death globally [1]. Heart disease is projected to remain the principal cause of death by 2030 [2], hence the significance of its early diagnosis and detection. Fortunately, several prophylactic strategies have been confirmed to lower the incidence of adverse cardiovascular events. Lifestyle modification and pharmacological interventions, including the administration of Statins (HMG-CoA reductase inhibitors), are examples of preventive approaches. Having access to effective prophylactic options, we feel obligated to determine risk assessment methods to achieve timely interventions.

Several risk factors have been proven valuable in CAD risk assessment, consisting of old age, male gender, specific ethnic groups, positive family history of CAD, sedentary lifestyle, inappropriate diet, smoking, obesity (controversial), hypertension, diabetes mellitus, dyslipidemia, and other metabolic or systemic disturbances with coronary artery involvement [3]. With outstanding advances in cardiac imaging techniques in recent years, several investigations were conducted to identify the application of new imaging biomarkers in the early diagnosis of at-risk populations to design preventive and management strategies [4].

Parallel to the advancement in technology, the application of multiplanar and intravascular imaging, including electrocardiogram (ECG)-gated CT and MRI, intravascular ultrasonography, and optical coherence tomography (IVOCT) in atherosclerotic plaque characterization, risk assessment, and designating an appropriate plan have developed. IVOCT is a novel CT-based method with limited application in clinical practice due to the clinicians’ unawareness of image interpretation and insufficient literature to develop an algorithmic approach. Active research is ongoing in the engineering and medical field to obtain more benefits from the use of technology in CAD management [5].

Among imaging indicators of cardiovascular health, coronary artery calcification, an independent predictor of cardiac health and adverse events, is used in standard clinical practice [6,7,8,9]. Coronary artery calcification quantification could be successfully performed utilizing computed tomography (CT).

It is detectable by different protocols, including ECG-gated calcium-scoring cardiac CT as the gold standard of care, followed by non-contrast chest CT. Since the publication of Agatston et al. in 1990 [10], who introduced the well-known Agatston method for coronary calcification measurement, multiple investigations have been conducted to seek measurement methods and their role in predicting outcomes. Calcium volume, mass, and density scores have been introduced with a description of the strengths and limitations of each technique [11,12].

Although ECG-gated CT is the standard method of measuring the coronary artery calcium score (CACS), ionizing radiation is still a concern in that while the average radiation dose is 1–2 mSv in most methods, doses of up to 10 mSv have also been reported [13,14,15].

Researchers have introduced various cardiovascular risk assessment methods for optimal CACS measurement with reduced radiation doses. Indeed, recent years have witnessed the emergence of novel reconstruction techniques, including iterative reconstruction (IR) and tube current-reduction methods, for CACS measurement in different populations [16,17,18]. Nonetheless, the literature has a dearth of studies employing different techniques to test the hypothesis that coronary artery calcification may be detectable by reduced radiation doses [19].

Accordingly, the present study investigated IR and Hounsfield unit (HU) threshold modification with the application of a low-dose 80 kVp protocol in ECG-gated coronary calcification imaging and compared the calculated CACS between these techniques and the standard 120 kVp filtered back projection (FBP)-reconstructed method.

## 2. Materials and Methods

The study protocol was approved by the institutional ethics committee and conducted in accordance with the Declaration of Helsinki.

### 2.1. Patients

Initially, outpatients referred to our center for coronary artery CT angiography from June through October 2021 were enrolled in the study. Patients with severe arrhythmias, coronary artery bypass grafts, coronary stents, prosthetic valves, pacemakers, or implantable cardioverter defibrillators were excluded. Informed consent was taken from each of the participants beforehand. Finally, 107 (mean ± standard deviation (SD) of age = 58.0 ± 10.6 y, 55.1% male) patients were included in data collection.

### 2.2. Data Collection

Patient preparation: The entire study population followed the institutional protocol for examination preparation: 4 h of fasting, consuming routine cardiovascular drugs, and taking a safe tranquilizer (10 mg of oxazepam) the night before the examination in case of anxiety. On the examination day, after routine heart rate (HR) evaluation, all patients with HR > 70 bpm received an oral β-blocker (50 mg of metoprolol) and were monitored every 30 min for rate control. Metoprolol was repeated in the event of poor HR control every 30 min (full dose = 200 mg). A vasodilator (0.4 mg of sublingual nitroglycerin pearl) was administered just before imaging to enhance coronary artery diameter, the number of well-visible branches, image quality, and the diagnostic accuracy of coronary CT angiography.

Image acquisition: All images were acquired using a 384-detector row dual-source scanner (SOMATOM Force, Siemens Healthineers, Erlangen, Germany). Each patient underwent a non–contrast-enhanced ECG-gated CT scan for CACS calculation twice.

a. Standard protocol: Prospective ECG-gated imaging was performed by applying a tube voltage of 120 kVp with automated tube current modulation. Spiral high-pitched (>3.2) tomography at the best diastolic point with 3 mm slice thickness and without gaps was carried out, covering the craniocaudal scan length from the bifurcation of the trachea to the left lateral pleural recess level. The FBP method was utilized for reconstruction.

b. Low-dose method: All the parameters were the same as those in the standard protocol except the tube voltage: a set of images was acquired with a tube voltage of 80 kVp. For reconstruction, in addition to the FBP, the new sinogram-affirmed iterative reconstruction (SAFIRE) algorithm was employed with different strength levels (1, 3, and 5). In this method, there are two correction loops. In the first loop, a ray tracing (reprojection) is applied to the FBP-reconstructed image in the raw data space. A new sinogram is obtained by utilizing the differences between the measured and the newly calculated projections. In the second loop, the image from the first loop is reprojected. Then, consecutive iterations correct the acquired projections. When the noise level of the acquired image approximates that of the selected iteration, the loop ends. The SAFIRE algorithm has five iteration levels that differ in the filtering intensity. A higher SAFIRE level provides smoother images with lower noise.

Patients’ classification: The study population was divided into two groups based on body mass index (BMI), composed of BMI < 30 kg/m^2^ and BMI ≥3 0 kg/m^2^, and three groups according to HR, consisting of HR < 60 bpm, HR = 60–75 bpm, and HR > 75 bpm.

CACS calculation: The calcium volume score was utilized for CACS calculation. This method encompasses all voxels with HU ≥ 130, with the score estimated by multiplying the number of voxels by their volumes [20]. The CACS was estimated for each set of images and the FBP-reconstructed 80 kVp series by changing the HU threshold of 130. The HU thresholds of 150, 170, and 190 were applied, and the CACS was calculated separately. The measurements were performed by two expert technologists with over 5 years of experience in coronary artery calcification imaging, and the intra- and interobserver variabilities were reported.

### 2.3. Classification of CACS

According to the available literature on cardiovascular risk assessment, six CACS groups were considered: 0, 1–10, 11–100, 101–400, 401–1000, and >1000. The agreement of the calculated CACS with the standard method was evaluated in the groups separately.

### 2.4. Statistical Analysis

Data analysis was conducted using IBM SPSS, version 23.00 (USA). Continuous and categorical variables were reported as mean ± SD and frequencies (percentages), respectively. Considering the FBP-reconstructed 120 kVp method as the standard CACS measurement, the reduced-dose technique results were compared with those from the standard protocol. The intraclass correlation coefficient (ICC) was utilized to determine the agreement between the results. ICC estimates with 95% confidence intervals (CIs) were reported based on mean rating, absolute agreement, and the two-way mixed-effects model. The ICC values were interpreted as <0.5 (poor agreement), 0.5–0.75 (moderate agreement), 0.75–0.9 (good agreement), and >0.9 (excellent agreement) [21]. *p* values < 0.05 were considered statistically significant.

## 3. Results

The present study enrolled 107 patients (mean ± SD of age = 58.0 ± 10.6 y, 55.1% male). The patients’ general characteristics and scan dose parameters are summarized in Table 1. The mean ± SD of the effective radiation dose was 0.60 ± 0.29 mSv in the 120 kVp scans and 0.21 ± 0.11 mSv in the 80 kVp scans, representing a 64% reduction in the effective radiation dose in the 80 kVp protocol.

The mean ± SD of CACS was 162.6 ± 302.4 in the standard images and 245.8 ± 421.3 in the FBP-reconstructed 80 kVp images. The mean ± SD of CACS in the IR 80 kVp series was 216.5 ± 381.5, 194.3 ± 350.1, and 162.6 ± 300.6 using the SAFIRE algorithm with strength levels of 1, 3, and 5, respectively. The inter- and intraobserver reliabilities were excellent (ICC > 0.9, *p* values < 0.05) for all the protocols.

**Figure 1:**
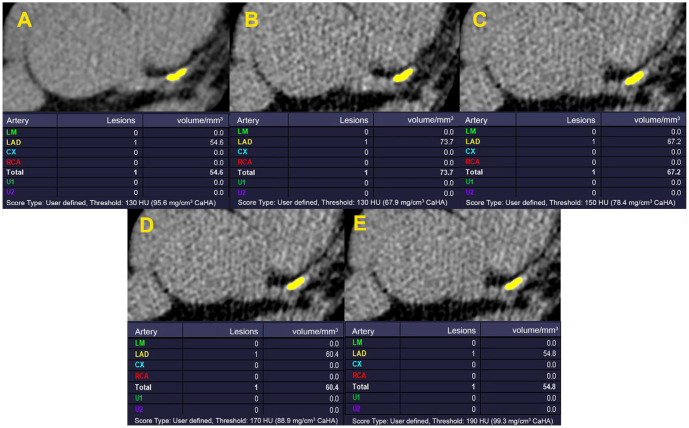
Comparison of the calculated CACS in standard (120 kVp FBP-reconstructed protocol with HU threshold: 130). (**A**) with 80 kVp FBP-reconstructed method applying HU thresholds: 130, 150, 170, and 190 (**B**–**E**). CACS was calculated for a proximal LAD artery lesion. Despite negligible visual differences in plaque shape, by increasing the HU threshold, the deviation of the calculated CACS from the standard image (**A**) declines. The nearest calculated CACS to the standard image is observed on image (**E**) at the HU threshold of 190. CACS: Coronary artery calcium score; FBP: filtered back projection; HU: Hounsfield unit; LAD: left anterior descending.

**Figure 2:**
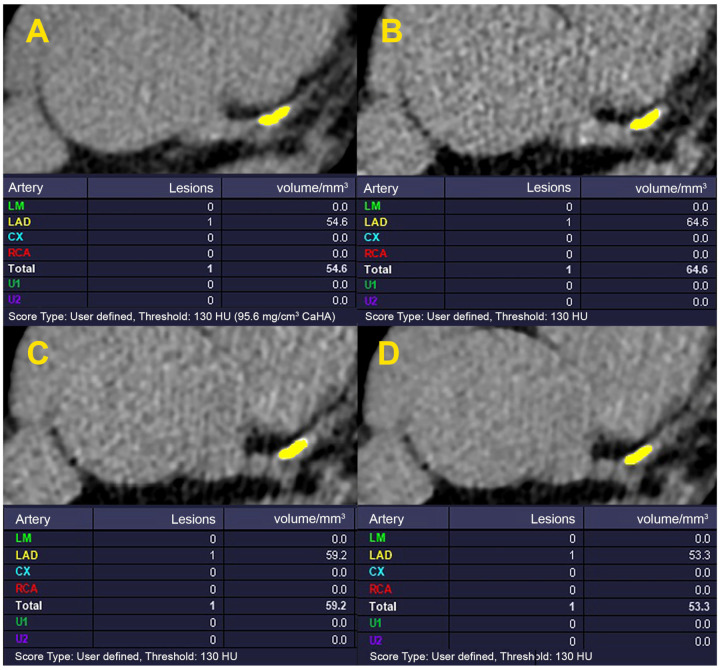
The CACS measurement in standard (120 kVp FBP-reconstructed protocol with HU threshold: 130) (**A**) and IR image sets with strength levels, 1, 3, and 5, and an HU threshold of 130 (**B**–**D**). CACS was calculated for the same lesion in Figure 1. Despite negligible visual differences in plaque shape, by increasing the IR strength level, the deviation of the calculated CACS from the standard image (**A**) decreases. The nearest calculated CACS to the standard image is observed on image (**D**) at the IR strength level: 5. CACS: Coronary artery calcium score; FBP: filtered back projection; HU: Hounsfield unit; IR: iterative reconstruction.

In each method of calcium scoring, the analysis was performed for different CACS groups. The agreement of all CACSs derived from the 80 kVp protocol (FBP-reconstructed with an HU threshold of 130, 150, 170, and 190, and IR with different strength levels at HU threshold of 130) with the standard protocol is depicted in Table 2 (Figure 1 and Figure 2). Despite insignificant visual differences noted in plaque shape, by increasing the HU threshold or IR strength level, the deviation of the calculated CACS from the standard protocol decreased. The closest measures to the standard method were observed in the HU threshold of 190 and IR strength level of 5.

All the calculated CACSs for the 80 kVp images demonstrated excellent agreement with the standard protocol in all the BMI and HR groups (ICC = 0.95–0.99 for the BMI groups and 0.95–1 for the HR groups).

Applying the 190 HU threshold in an 80 kVp image set resulted in the reclassification of eight patients (7.5%), five of whom had a BMI < 30 kg/m^2^, two had an HR < 60 bpm, and seven had an HR = 60–75 bpm. Furthermore, with the utilization of IR, the SAFIRE algorithm with a strength level of 5 in 80 kVp images, nine patients (8.4%) were reclassified to a different CACS risk group (Table 3). All of them were reclassified in one upper or lower class and had a CACS < 100. Eight of the nine patients had a BMI < 30 kg/m^2^, four had an HR < 60 bpm, and six had an HR = 60–75 bpm.

## 4. Discussion

In this study, we compared the CACS calculated according to the standard FBP-reconstructed 120 kVp protocol with an HU threshold of 130 and CACS derived from 80 kVp images applying either IR (with different strength levels and HU threshold = 130) or FBP (with different HU thresholds). Our results revealed that the CACS obtained via the reduced kVp technique had excellent agreement with the standard protocol, particularly when IR was applied with a strength level of 5 or FBP with an HU threshold of 190.

Our study is among the few in vivo studies to compare dose-reduction techniques for CACS measurement in cardiovascular risk assessment with the standard method. Reducing ED in coronary artery calcification scoring has been a hot topic recently. These techniques comprise two principal components: (1) lowering the tube voltage, tube current, or both, and (2) using complementary post-acquisition methods, such as IR algorithms and HU threshold modification, to compensate for possible image decay as a result of lower radiation [22,23]. Studies are controversial in this regard since some have claimed high accuracy compared to standard methods, whereas others have reported low accuracy and a failure to detect calcification in low-dose protocols. Therefore, there is a need for valid and standardized studies to compare the different combinations of dose reduction techniques to determine the best possible protocol for CACS calculation [24,25,26,27].

Our results conform with previous studies using the same IR algorithms with tube current/voltage reduction. A previous phantom study stated that CACS accuracy would not decline if the adaptive iterative dose reduction three-dimensional algorithm is applied. They proved their finding even with an ED reduction of up to 80% of the standard protocol [28]. In another investigation, a low tube-current (75, 50, and 25% of the original value) simulation was performed utilizing artificial noise. The authors applied FBP and adaptive iterative dose-reduction the -dimensional (mild, standard, and strong) reconstruction methods. They reported that an up to 75% reduction in the tube current is attainable if we use the adaptive iterative dose reduction three-dimensional (mild) algorithm for reconstruction [19]. In a subsequent outstanding report, the investigators included 200 cases prospectively and, similar to our research, acquired two image sets for CACS measurement: one in the standard and the other in reduced dose via tube current reduction. Likewise, FBP and IR were applied for reconstruction, and the calculated CACS were compared. They reported a 74% radiation dose reduction when performing reduced-dose image acquisition and IR [29]. All described studies are remarkable reports on IR application in combination with tube current modification to reduce the radiation dose for CACS measurement.

On the other hand, there are investigations on low-voltage imaging protocols for CACS assessment. In a previous large-scale study, the authors compared CACS derived from 120 and 80 kVp protocols and risk reclassification incidence. They particularly examined the differences in revealing the zero calcium score. Their investigation revealed a good correlation of calculated CACS between 120 and 80 kVp protocols with apparent CACS underestimation in the low-voltage method. Moreover, they concluded that low-voltage CT may influence the risk estimation with a special impact on the zero calcium predictive value [30]. Similarly, we worked on the low-voltage 80 kVp image sets, but the power of our work was applying post-processing techniques, IR with variable strength levels, and threshold modifications. We revealed that the agreement between CACS derived from low-voltage image sets, and that the standard method remarkably improves when applying IR (strength level of 5) and increasing the density threshold from 130 HU to 190 HU.

Another valuable study was conducted on 103 patients at 120, 100, 80, and 70 kVp protocols and examined a novel kVp-adapted threshold determination method for CACS calculation. They introduced a mathematical Agatston-based method for the calculation of kVp-adapted thresholds. They revealed that as the tube voltage decreases, the thresholds increase and proved their method accurate for CACS measurement with up to 80% reduction in radiation dose [31]. Outstandingly, we demonstrated similar findings with the application of 130, 150, 170, and 190 HU thresholds for calcium volume score measurement.

There are controversial reports about the results of utilizing IR algorithms for CACS calculation. In a previous study by Tesche et al. on 60 patients, a 100 kVp protocol with tin filtration was applied. The raw images were reconstructed by FBP and iterative algorithm (strength levels of 3 and 5). The authors concluded that in comparison to the FBP, the IR method is associated with lower image noise and Agatston scores and emphasized that the latter finding may influence cardiovascular risk classification [32]. In another investigation, which included both phantom and in vivo data, the accuracy of IR algorithms was compared to that of the FBP technique. The authors revealed that IR algorithms do not influence the reproducible Agatston CACS measures and cardiac risk classification [33]. To our knowledge, our study is one of the first investigations that utilizes the combination of low-voltage image acquisition protocol and SAFIRE with three different strength levels and compares the results with the standard method. Remarkably, we noticed further agreement between low-voltage IR-derived CACS and standard 120 kVp FBP-reconstructed-derived calcium measures with increasing the IR strength levels. However, more investigations are needed to establish an accurate conclusion.

Recently, Luhur et al. [34] showed perfect accuracy with a 25% dose reduction by applying a 75% tube current reduction and an IR algorithm. They stated that voltage or current reduction significantly increased image noise, which was more pronounced in higher Agatston scores. The increased noise (when using a 75% reduction) not only led to the systematic overestimation of the Agatston score due to the overestimation of the size of lesions but also impacted cardiovascular risk stratification. Consequently, we chose a 33% reduction in the tube voltage to obtain acceptable image quality. Matsuura et al. [27] compared CACS to FBP reconstruction at the conventional tube current and CACS to hybrid IR at a lower tube current in 77 patients and confirmed that CACS was comparable between the two protocols.

Despite the recent advancements in CT scanner technology, in hardware and software, which have resulted in remarkably lower radiation doses, multiple investigations evaluated the dose-reduction methods for CACS imaging. However, these methods have not received acceptance for routine practical use. We suppose that it is due to the fact that the accuracy and value of the CACS derived from standard 120 kVp FBP-reconstructed images were examined enormously, which reassures the imaging professionals to be on the correct way. Any protocol substitution needs to be sufficiently validated by experts before entering the routine daily practice.

We observed reclassification in eight (7.5%) and nine (8.4%) patients in the two protocols with maximal agreement with the standard protocol. In the IR 80 kVp method with a threshold of 130 HU, none of the nine reclassifications altered the management strategy. In other words, all the reclassified patients were still in the medical treatment or coronary artery angiography group as their next step. Similarly, when we employed FBP-reconstructed 80 kVp with a threshold of 190 HU, none of the reclassifications resulted in a change in the patient plan. These findings support the investigated methods as acceptable and possible substitutes for the standard protocol.

Our results demonstrated an excellent correlation regarding CACS between reduced-dose methods and standard images in all HR and BMI groups. Most reclassification cases were in the BMI < 30 kg/m^2^ and HR < 75 bpm groups. A previous study utilized low-voltage methods associated with threshold modification and reported no reclassification for the 80 kVp and 70 kVp scans when BMI < 24 kg/m^2^ and <21 kg/m^2^, respectively [35]. Another investigation recommended BMI-adapted peak tube voltage in 120 kVp, 80 kVp, and 70 kVp image sets for subjects with BMI ≥ 25 kg/m^2^, <24 kg/m^2^, and <21 kg/m^2^, respectively [31]. The influence of HR on CACS was investigated in a phantom study with four different CT machines, and the results showed increased Agatston scores and high-calcium contents in higher HR [36]. Accordingly, we suppose that BMI and HR should be considered when reduced-dose methods are planned. We also believe that more research applying different low-dose protocols in various BMI and HR categories is needed to determine the most precise CACS.

We chose the calcium volume score for the CACS measurement instead of the well-known Agatston score. It is believed that the calcium volume score is the most reproducible method [20]. Moreover, contrary to the Agatston method, it does not take a density factor in the calculation, which is more logical considering the pathologic basis of atherosclerosis [4]. We observed excellent intra- and interobserver reliability for all measured scores. Our aim was to assess the accuracy of CACS; thus, we applied the most robust method (calcium volume score). This method may have limitations in follow-up measures due to its susceptibility to the partial volume effect. In different examinations, the position of the plaque in the axial slice changes, posing some challenges.

To our knowledge, this is the first investigation to examine low-kVp coronary artery calcification images acquired using a 384-detector row dual-source scanner (SOMATOM Force, Siemens Healthineers, Erlangen, Germany). The acquisition parameters vary among scanners; different protocols should, therefore, be evaluated in various settings.

The acquisition of both low- and high-kVp images in a single patient population provides reliable findings since each patient is compared with themselves, serving as the best control. However, ionizing radiation exposure causes concerns regarding the patients’ safety. In the present investigation, we performed an extra CACS imaging using the 384-detector row dual-source scanner (SOMATOM Force, Siemens Healthineers, Erlangen, Germany), which imposed a negligible extra dose of 0.21 mSv ± 0.11 on each patient. Previous investigations established that radiation-related cancer risk is a concern at doses above 100 mSv [37], which is extremely far from even the total cumulative radiation dose that our subjects received (0.60 mSv ± 0.29 and 0.21 mSv ± 0.11, less than one mSv). In radiation doses of 10–100 mSv, there is a controversy about the cancer risk [38]. However, in radiation doses <10 mSv, there is no supporting data of increased cancer risk [39]. Therefore, we believe that the extra radiation dose that our study subjects received is negligible. Although we were sure about this issue, we explained it comprehensively to individual cases and obtained written informed consent. Furthermore, the ethics committee confirmation was achieved prior to the study’s conduction.

Our findings, albeit valuable in the cardiovascular imaging era, should be interpreted in light of some limitations. First and foremost, despite our comprehensive explanations of the procedure and the negligible additional dose, a few patients consented to double exposure, precluding the expansion of the sample size. Additionally, the fact that the mean BMI of the study population was >30 kg/m^2^ implied that our volunteers were more from overweight and obese populations, preventing the analysis of BMI adaptation for each protocol. Further multicentric studies can overcome these drawbacks.

## 5. Conclusions

In the present study, with a 33% reduction in the tube voltage in coronary artery calcification imaging, a 64% reduction in ED occurred. The application of IR or threshold modification is associated with the acceptable accuracy of estimated CACS, while negligible cardiovascular risk reclassification is observed.

## Figures and Tables

**Table 1:** General characteristics of the study population and scan-dose parameters.

Characteristic	Measure
Age (y) (mean ± SD)	58.09 ± 10.60
Gender, male, *n* (%)	59 (55.1%)
Weight (kg) (mean ± SD)	81.08 ± 13.70
Height (m) (mean ± SD)	1.68 ± 9.00
Body mass index (kg/m^2^)	33.64 ± 4.50
Average heart rate (bpm)	63.92 ± 7.41
DLP (mGy·cm) in the reduced-dose method and for the full scan length (mean ± SD)	15.77 ± 8.69
Effective dose (mSv) in the reduced-dose method and for the full scan length (mean ± SD)	0.21 ± 0.11
DLP (mGy·cm) in the standard method and for the full scan length (mean ± SD)	44.51 ± 21.67
Effective dose (mSv) in the standard method and for the full scan length (mean ± SD)	0.60 ± 0.29

y: Year; SD: standard deviation; n: number; kg: kilogram; m: meter; bpm: beats per minute; mGy: milligray; cm: centimeter; mSv: millisievert; DLP: dose length product.

**Table 2:** Measured CACS in IR and threshold modification methods, and their agreement with the standard protocol. ICC calculated in comparison to FBP-reconstructed 120 kVp with an HU threshold of 130.

	CACS Group	ICC	Agreement	*p* Value
FBP-reconstructed 80 kVp with a threshold of 130 HU	0	0.15	Poor	0.3
	1–10	0.29	Poor	0.04
	11–100	0.67	Moderate	<0.001
	101–400	0.48	Poor	0.003
	>400	0.96	Excellent	<0.001
FBP-reconstructed 80 kVp with a threshold of 150 HU	0	0.36	Poor	0.1
	1–10	0.37	Poor	0.09
	11–100	0.79	Good	<0.001
	101–400	0.78	Good	<0.001
	>400	0.97	Excellent	<0.001
FBP-reconstructed 80 kVp with a threshold of 170 HU	0	0.60	Moderate	0.009
	1–10	0.53	Moderate	0.03
	11–100	0.89	Good	<0.001
	101–400	0.87	Good	<0.001
	>400	0.97	Excellent	<0.001
FBP-reconstructed 80 kVp with a threshold of 190 HU	0	0.84	Good	<0.001
	1–10	0.65	Moderate	0.02
	11–100	0.94	Excellent	<0.001
	101–400	0.92	Excellent	<0.001
	>400	0.97	Excellent	<0.001
IR 80 kVp with a strength level of 1 and threshold of 130 HU	0	0.18	Poor	0.3
	1–10	0.36	Poor	0.02
	11–100	0.76	Good	<0.001
	101–400	0.74	Moderate	<0.001
	>400	0.97	Excellent	<0.001
IR 80 kVp with a strength level of 3 and threshold of 130 HU	0	0.36	Poor	0.1
	1–10	0.48	Poor	0.01
	11–100	0.88	Good	<0.001
	101–400	0.85	Good	<0.001
	>400	0.98	Excellent	<0.001
IR 80 kVp with a strength level of 5 and threshold of 130 HU	0	0.58	Moderate	0.01
	1–10	0.67	Moderate	0.006
	11–100	0.96	Excellent	<0.001
	101–400	0.93	Excellent	<0.001
	>400	0.99	Excellent	<0.001

CACS: Coronary artery calcium score; IR: iterative reconstruction; ICC: intraclass correlation coefficient; FBP: filtered back projection; HU: Hounsfield unit.

**Table 3:** CACS group reclassification.

CACS in Standard Protocol	Calculated CACS in FBP-Reconstructed 80 kVp with an HU Threshold of 190					
CACS Group	0	1–10	11–100	101–400	>400	Total
0	26					26
1–10	3	12	3			18
11–100		1	25			26
101–400			1	22		23
>400					14	14
Total	29	13	29	22	14	107
	**Calculated CACS in IR 80 kVp with the Strength Level of 5 with an HU Threshold of 130**					
**CACS Group**	**0**	**1–10**	**11–100**	**101–400**	**>400**	**Total**
0	23	3				26
1–10		14	4			18
11–100		1	24	1		26
101–400				23		23
>400					14	14
Total	23	18	28	24	14	107

The yellow color demonstrates the patients with no change in the CACS class. CACS: Coronary artery calcium score; IR: iterative reconstruction; HU: Hounsfield unit; FBP: filtered back projection.

## Data Availability

The datasets generated during the current report are available from the corresponding author upon reasonable request.
